# Lung ultrasound for the diagnosis of pulmonary atelectasis in both adults and pediatrics: A protocol for systematic review and meta-analysis

**DOI:** 10.1097/MD.0000000000031519

**Published:** 2022-11-18

**Authors:** Wenlong Liu, Xu Zhang, Kai Liu, Zhongjing Kang

**Affiliations:** a Ultrasonic Center, the Affiliated Hospital of Guizhou Medical University, Guizhou, China; b Department of Medical Records, First Affiliated Hospital, Heilongjiang University of Chinese Medicine, Heilongjiang, China; c Endoscopy Room, Shengjing Hospital Affiliated to China Medical University, Liaoning, China; d Department of Radiology, Songshan General Hospital, Chongqing, China.

**Keywords:** diagnosis, lung ultrasound, meta-analysis, pulmonary atelectasis, systematic review

## Abstract

The use of lung ultrasound for the diagnosis of pulmonary atelectasis remains controversial. Therefore, we performed a protocol for systematic review and meta-analysis to evaluate the diagnostic accuracy of lung ultrasound for the diagnosis of pulmonary atelectasis both in adults and pediatrics.

**Methods:**

A comprehensive search of several databases from 1966 to October 2022 will be conducted. The databases include Ovid Medline In-Process & Other Non-Indexed Citations, Ovid MEDLINE, Ovid EMBASE, Ovid PsycINFO, Ovid Cochrane Central Register of Controlled Trials, Ovid Cochrane Database of Systematic Reviews, and PubMed. After screening and diluting out the articles that met inclusion criteria to be used for statistical analysis, the pooled evaluation indexes including sensitivity and specificity as well as hierarchical summary receiver operating characteristic curves with 95% confidence interval were calculated. All statistical analyses were calculated with STATA, version 12.0 (StataCorp, College Station, TX).

**Result:**

We will synthesize the current studies to evaluate the diagnostic accuracy of lung ultrasound for the diagnosis of pulmonary atelectasis.

**Conclusion:**

The result of this review will provide more reliable references to help clinicians make decisions for the diagnosis of pulmonary atelectasis.

## 1. Introduction

Pulmonary atelectasis is the loss of lung volume resulting from bronchial obstruction (obstructive atelectasis) or from parenchymal compression (nonobstructive atelectasis).^[1,2]^ Atelectasis produces a ventilation–perfusion mismatch, an intrapulmonary shunt, an increase in pulmonary vascular resistance and arterial hypoxemia.^[3,4]^ Furthermore, loss of aerated lung may increase the risk of pneumonia and ventilator-induced lung injury by overstretching of the aerated lung

Previously, the diagnosis of lung consolidation or atelectasis depended mainly on X-ray film or computed tomography^[5,6]^, however, these examinations are clinically impractical, time-consuming, expensive and harmful exposition to X-ray, especially for severely ill patients. The recurrence of life-threatening pulmonary complications in this very fragile population, requiring continuous radiological exams, highlights the urgent need to identify alternative, noninvasive, reproducible, and radiation-free diagnostic tools.^[7]^

Ultrasound imaging has emerged as a reliable technique for the evaluation of various lung diseases and has become a first-line tool in critical and emergency care settings, with international consensus guidelines for its use.^[8,9]^ Lung ultrasound seems to be a useful tool for diagnosing and evaluating pulmonary atelectasis, and more generally, alveolar consolidation. In patients with total or nearly total opacification of the hemithorax on a chest X-ray study, ultrasound imaging shows high sensitivity in detecting pleural and parenchymal lesions making it possible to differentiate between pleural effusion and consolidation.^[10]^ Lung ultrasound was considered not possible in the past, for the presence of air in the normal lungs and the bony thoracic cage.^[11]^ However, the use of flexible probes that can explore the hidden lung regions and the analysis of different ultrasound artifacts in the pathological and normal lung have overcome these limitations. However, the use of lung ultrasound for the diagnosis of pulmonary atelectasis remains controversial. Therefore, we performed a protocol for systematic review and meta-analysis to evaluate the diagnostic accuracy of lung ultrasound for the diagnosis of pulmonary consolidation or atelectasis both in adults and pediatrics.

## 2. Methods

### 2.1. Study registration

The protocol was designed in accordance with the Preferred Reporting Items for Systematic Reviews and Meta-Analysis guidelines extension for reporting systematic review protocols (PRISMA-P).^[12]^ The review protocol was registered with the International Prospective Register of Systematic Reviews (PROSPERO), registration number (CRD42020162676). Ethical approval was not required for this study as all the research materials are derived from published studies.

### 2.2. Inclusion criteria

Inclusion criteria should follow all items: clinical studies involved adults and pediatrics with pulmonary atelectasis; 1 study used imaging modalities including CT and lung ultrasound simultaneously for the detection of pulmonary atelectasis; study compared the diagnostic value of CT and lung ultrasound; studies provided original diagnostic data (true positive, false positive, false negative, and true negative) or can be calculated using enough evidence; studies presenting the most data values was included this statistical analysis if literatures contain overlapping data. Exclusion criteria comprised: letters, conference summary, meeting abstract, commentary and other no full-text studies; animal and cadaver experiments; and articles presenting non original diagnostic data directly or no enough evidence to calculate diagnostic data indirectly.

### 2.3. Search methods

A comprehensive search of several databases from 1966 to October 2022 will be conducted. The databases includes Ovid Medline In-Process & Other Non-Indexed Citations, Ovid MEDLINE, Ovid EMBASE, Ovid PsycINFO, Ovid Cochrane Central Register of Controlled Trials, Ovid Cochrane Database of Systematic Reviews, and PubMed. Search strategy for PubMed was shown in Table 1. Two authors will independently draft and carry out the search strategy. In addition, we manually retrieve other resources, including the reference lists of identified publications, conference articles, and gray literature. The key terms used for the search are “lung ultrasound,” “pulmonary atelectasis” and “lung consolidation.” The selection process of eligible papers is shown in a PRISMA flow diagram (Fig. 1).

**Table1:** Search strategy for PubMed.

#1	Ultrasonography[MeSH Terms]
#2	Ultrasonography[Title/Abstract]
#3	Ultrasonics[Title/Abstract]
#4	Diagnostic imaging[Title/Abstract]
#5	Ultrasound[Title/Abstract]
#6	Echography[Title/Abstract]
#7	Ultrasound*[Title/Abstract]
#8	Ultrasonic[Title/Abstract]
#9	Echotomography[Title/Abstract]
#10	#1 OR #2 OR #3 OR #4 OR #5 OR #6 OR #7 OR#8 OR#9
#11	Pulmonary Atelectasis[MeSH Terms]
#12	Pulmonary Atelectasis[Title/Abstract]
#13	Atelecta*[Title/Abstract]
#14	Pulmonary consolidation[Title/Abstract]
#15	lung consolidation[Title/Abstract]
#16	#11 OR #12 OR #13 OR #14 OR #15
#17	#10 AND #16

**Figure. 1.**
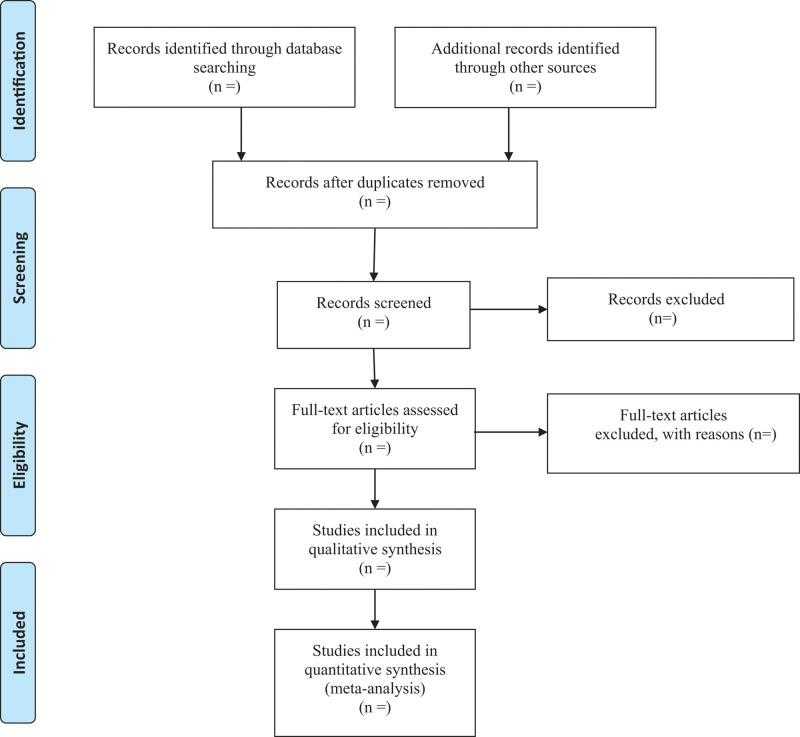
Flow diagram of study selection.

### 2.4. Data extraction and quality assessment

First, we will extract and record the first author’s name, year of publication, study design, group information, age, gender, dropouts, sample size, duration of intervention and outcome from the studies that met the inclusion criteria. We will contact the corresponding authors for additional information if necessary. Second, diagnostic data including true positive, false positive, false negative, and true negative were extracted. To reduce potential bias, all targeted data were extracted into a standardized form by 2 independent and blinded researchers. We used a quality assessment tool (QUADAS-2).^[13,14]^ to evaluate the methodological quality of the included studies. This tool consists of 11 items, and if the included study meets 1 item, 1 score will be given. The quality of each included studies was assessed by 2 independent and blinded researchers. Inconsistencies between researchers were resolved by consensus.

### 2.5. Data analysis

A bivariate random-effects model was applied to derive summary estimates of the diagnostic value by merging the following pooled outcome estimates: sensitivity, specificity, and hierarchical summary receiver operating characteristic curves.^[15]^ Heterogeneity between studies was evaluated using Cochran’s Q test (*P*<.05 indicating the presence of heterogeneity). Subgroup analysis will be performed to reduce the heterogeneity and ensure the accuracy of results. Deeks’ funnel plot asymmetry test^[16]^ was omitted to assess publication bias according to the PRISMA-DTA. All statistical analyses were calculated with STATA, version 12.0 (StataCorp, College Station, TX). A 2-sided *P*<.05 were considered as significant

### 2.6. Assessment of quality of evidence

We will use the Grading of Recommendations Assessment, Development, and Evaluation (GRADE) to assess the results.^[17]^ In the GRADE system, the quality of evidence will be categorized into 4 levels: high, moderate, low, and very low quality

## 3. Discussion

Pulmonary atelectasis consists of pulmonary collapse, accompanied by hypoventilation; it can affect a lobe, segment, or all of the lung, resulting in a decrease in the ventilation/perfusion ratio. Its incidence varies according to the age and characteristics of the population studied. Generally, the diagnosis of pulmonary atelectasis is based on history, clinical and chest X-ray or CT findings while ultrasound could not be used in lung disease diagnostics.^[18]^ Recently, ultrasound has been used for the diagnosis of many kinds of lung conditions, but few studies have investigated ultrasound for the diagnosis of pulmonary atelectasis.^[19,20]^ To the best of our knowledge, this is the first meta-analysis to evaluate the accuracy of lung ultrasound in detection of pulmonary atelectasis. We hope that the result of this review will provide more reliable references to help clinicians make decisions when dealing with pulmonary atelectasis.

## Author contributions

**Conceptualization:** Xu Zhang.

**Data curation:** Kai Liu.

**Investigation:** Kai Liu.

**Writing – original draft:** Wenlong Liu.

**Writing – review & editing:** Zhongjing Kang.
